# Short-term and long-term associations between household wealth and physical growth: a cross-comparative analysis of children from four low- and middle-income countries

**DOI:** 10.3402/gha.v8.26523

**Published:** 2015-02-05

**Authors:** Aditi Krishna, Juhwan Oh, Jong-koo Lee, Hwa-Young Lee, Jessica M. Perkins, Jongho Heo, Young Sun Ro, S.V. Subramanian

**Affiliations:** 1 Department of Social and Behavioral Sciences, Harvard T.H. Chan School of Public Health, Boston, MA, USA; 2 JW LEE Center for Global Medicine, Seoul National University College of Medicine, Seoul, Korea; 3 Department of Health Policy, Harvard University, Cambridge, MA, USA; 4 Public Health Joint Doctoral Program, San Diego State University & University of California, San Diego, CA, USA

**Keywords:** socioeconomic conditions, poverty, child nutrition, life-course epidemiology

## Abstract

**Background:**

Stunting, a form of anthropometric failure, disproportionately affects children in developing countries with a higher burden on children living in poverty. How early life deprivation affects physical growth over various life stages is less well-known.

**Objective:**

We investigate the short- and long-run associations between household wealth in early life with physical growth in childhood in four low- and middle-income countries to understand the persistent implications of early life conditions of poverty and resource constraints on physical growth.

**Design:**

Longitudinal study of eight cohorts of children in four countries – Ethiopia, India, Peru, and Vietnam (*n=*10,016) – ages 6 months to 15 years, using data from the Young Lives project, 2002–2009. Physical growth outcomes are standardized height-for-age z-scores (HAZ) and stunting. The key exposure is household wealth measured at baseline using a wealth index, an asset-based indicator. Covariates include child's age and sex, caregiver's educational status, household size, and place of residence.

**Results:**

Baseline wealth index is significantly associated with higher physical growth rates as suggested by higher HAZ and lower odds of stunting. We found these associations in all four countries, for younger and older cohorts and for children who experienced changes in living standards. For the older cohort, despite the timing of the first survey at age 7–8 years, which is beyond the critical period of 1,000 days, there are lasting influences of early poverty, even for those who experienced changes in wealth.

**Conclusions:**

Household wealth in early life matters for physical growth with conditions of poverty and deprivation influencing growth faltering even beyond the 1,000 days window. The influences of early childhood poverty, so prevalent among children in low- and middle-income countries, must be addressed by policies and programs targeting early life but also focusing on older children experiencing growth faltering.

Globally, 165 million children under the age of five are stunted (1). Nearly 90% of these children – 148 million – live in low- and middle-income countries (2). Stunting (low height-for-age), a marker of anthropometric failure, disproportionately affects children in poorer countries and from poorer households (2, 3). Social, economic, political, and environmental conditions operate through proximal factors such as food availability, access to health services, and hygienic provisions for water and sanitation. These resources then affect behaviors such as food preparation, breastfeeding, and management of infectious diseases, affecting physical growth (3–6). These factors are recognized as key determinants affecting more proximal causes of physical growth.

Several studies investigate the relationship between household social and economic conditions and stunting, finding strong correlations between conditions of household poverty and deprivation and children's growth and development in both developing and developed countries (7–12). Much of this work has been cross-sectional, examining contemporaneous associations between current poverty and stunting. Other studies use longitudinal data, following individuals over time to examine influences of poverty in early life on later life outcomes (13–18). Importantly, longitudinal studies are better able to causally link early socioeconomic conditions to later child growth because they can establish temporal precedence. However, due to the difficulty of measuring early life conditions and in following individuals over time, a limited number of studies consider the long-term effects of poverty in early life on later health outcomes (12). Indeed, only a few longitudinal studies of poverty and child growth and health exist in low- and middle-income countries; yet, these studies provide initial evidence that poorer households experience higher infant and child mortality, higher prevalence of anthropometric failure, and poorer child health and developmental outcomes along the life-course (19–23). Our study seeks to fill this gap in the literature by considering the lasting influences of household wealth on stunting in various stages of infancy, childhood, and adolescence, building on work looking at associations between poverty and physical growth at one life stage. In contrast to studies in only one context, this investigation includes cross-national comparisons for eight cohorts of children in four low- and middle-income countries.

In this study, we examine the influence of household wealth in early life (e.g. infancy and childhood) on later physical growth using the household wealth index as a composite measure of living standards. Using panel data representing eight cohorts from four low- and middle-income countries, we investigate the association between the wealth index at the beginning of data collection and physical growth at baseline and at three time points (infancy, childhood, and adolescence). Lastly, we examined the relationship between household wealth and physical growth in four countries with different social, cultural, economic, and political contexts to understand whether better living standards improve children's growth rates in diverse settings.

## Methods

### Study population

This study uses data from the Young Lives study, a longitudinal study of child health and well-being in four countries – Ethiopia, India, Peru, and Vietnam (24–28). The Young Lives study collected quantitative surveys of children, their household members, communities, and schools as well as qualitative interviews with a subset of respondents. The first round of quantitative data collection occurred in 2002 with two rounds of follow-up surveys in 2006–2007 and 2009. Young Lives was designed to follow two cohorts: a younger cohort born in 2000–2001 and an older one born in 1994–1995. Three rounds of data are currently available with two more projected for the future.

The sampling design for Young Lives was similar across the four countries. As the goal of the survey was to assess conditions of health and well-being among poorer children, the sampling methodology was geared to oversample poor populations, as defined by country staff (29). In each country, staff chose 20 sentinel sites in poor areas, enumerating all households with living children born between 2000 and 2001 and with children born between 1994 and 1995. From this list, they randomly chose 150 households (100 for the younger cohort and 50 for the older cohort) to participate in the study within each site. Households that refused to participate – less than 2% of the selected households – were replaced with other households from the list (24). Refusal rates were low in all four countries because the study used local staff, facilitating greater comfort between participants and enumerators (24). One child per household was chosen as the index child, resulting in 2,000 children surveyed for the younger cohort in each country and 1,000 children surveyed for the older cohort in each country. More information on the sampling strategies in each country can be found in Outes-Leon and Sanchez (30), Kumra (31), Escobal and Flores (32), and Nguyen (33). Attrition rates were notably low, ranging from 0.50 to 3.52%, compared to other longitudinal studies in similar contexts (34), and were similar across countries though younger cohorts had slightly higher attrition and both cohorts did so in Peru compared to other countries. However, due to the large sample size and the potential of attrition bias (34), only individuals with data for all rounds were used.

### Explanatory measure and covariates

Filmer and Pritchett developed the wealth index to measure living standards among households in developing countries because of poor availability of income, wealth, or expenditure data (35). The wealth index is a composite measure, developed through selection of key elements of living standards and refined through principal components analysis (35–37). It considers number of household members, ownership of material goods, housing quality, water and sanitation quality, and access to energy sources. Although the wealth index is not an adequate measure of consumption expenditure (38), it has been widely used as an indicator of living standards in low-resource settings (39). In the Young Lives study, a wealth index was constructed from three equally weighted components – a housing quality index, a services quality index, and a consumer durables index – and ranges from 0 to 1 (37). We multiplied the wealth index values by 100 for ease of interpretation. More information on construction of the wealth index is available in Table A1.

The baseline wealth index was measured at age 6–18 months for the younger cohort and age 7–8 years for the older cohort, representing living standards in infancy for the younger cohort and in childhood for the older cohort. In our analyses, we used the baseline wealth index as a continuous measure as well as a categorical measure, by creating wealth tertiles (very poor, poor, and less poor) from the baseline wealth index. In addition, we included a set of child-level and household-level covariates to account for possible confounding. These items included child's age and sex, caregiver's educational attainment classified into three categories (none, 5 or less years, or more than 5 years of schooling), household size (number of members), and place of residence (urban/rural). Nearly all caregivers (97.5%) were biological parents, of whom nearly all are mothers.

### Outcome measures

The key outcome of interest was physical growth measured as height-for-age with low height-for-age or stunting as a secondary outcome. Shorter stature is a reflection of chronic undernutrition leading to impaired physical development. Thus, low height-for-age is one measure of the effects of long-term nutritional deficiency on child growth and is linked with higher child morbidity and mortality as well as lasting physical, cognitive, and psychological impairments (3). Globally, low height-for-age is measured through standardized anthropometric data using WHO reference populations and operationalized into height-for-age z-scores (HAZ) and stunting, which is two standard deviations below the median HAZ for reference populations. For children under 5 years, the Multicentre Growth Reference Study was used as the reference population while data from the National Center for Health Statistics from 1977 merged with WHO data were used for older children (24, 40). For both cohorts, anthropometric data were obtained by trained enumerators at three time points – in infancy (6–18 months), early childhood (4–5 years), and late childhood (7–8 years) for the younger cohort; and in late childhood (7–8 years), early adolescence (11–12 years), and late adolescence (14–15 years) for the older cohort. Young Lives staff obtained weight, height, and length data for the younger cohort in round 1, in accordance with WHO recommendations about the appropriateness of length rather than height for children under age 2 years (41). In subsequent rounds, enumerators collected only weight and height of children. For weight, length, and height, two measurements were taken for each child. If the measurements reflected the same values, they were recorded. If agreement was not obtained between the two measurements, repeated measures were taken until complete consensus between measures was obtained. Final height and weight data in Young Lives data reflect reliable measurements taken by trained staff.

### Statistical models

Descriptive statistics were estimated for each country, pooling across rounds and cohorts. Mean HAZ and prevalence of stunting overall and by baseline wealth index category in each round, cohort, and country was also estimated. Ordinary least squares and logit regression models were used to assess associations between baseline wealth index and HAZ and stunting, respectively, as well as changes in associations over time. All models include survey and sentinel site fixed effects as well as individual-level random effects to adjust for correlated data and clustered standard errors (SEs) at the sentinel site level (42). Analyses were stratified by country and cohort. Models using HAZ as the outcome are presented in the main text, and results regarding stunting are presented in Appendix . Unadjusted models and adjusted models controlling for child's age and sex, caregiver's educational attainment, household size, and place of residence were used. Analyses were conducted in Stata version 13.1, R version 3.1.1, and R Studio version 0.98.1028.

### Sensitivity analyses

It is possible that the baseline wealth index represents a lifetime of deprivation affecting children over their life-course. This is a concern particularly for the Young Lives sample and for many poor children in low- and middle-income countries who may not experience any changes in living standards (43). For these children, it is more difficult to disentangle the influences of the baseline wealth index from living standards later in life. To address this possibility, we controlled for whether individuals had any change in wealth index tertiles over the three survey rounds – operationalized as either upward or downward mobility in tertiles – to compare the relationship between baseline wealth index and physical growth in the group who experienced wealth changes to the group of children who experienced no change in wealth index tertiles. Additional models included an indicator for whether children experienced any change in wealth index over all survey periods as well as interaction terms between the indicator and the continuous version of the baseline wealth index.

## Results

**Fig. 1:**
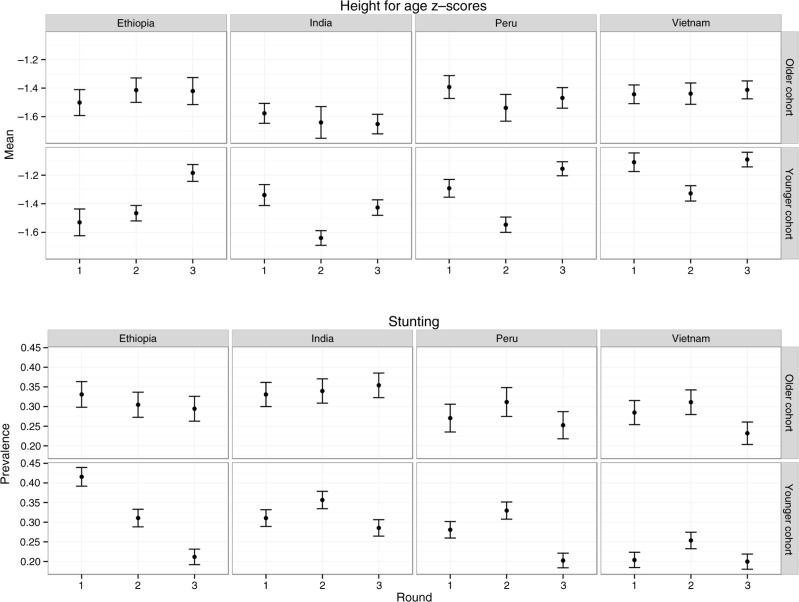
Mean height-for-age z-scores and prevalence of stunting over Young Lives surveys, by country and cohort.

Table 1 contains sample sizes by country and cohort with some variation due to attrition and missing data on key covariates. Mean HAZ and stunting prevalence for each country, survey round, and cohort are presented in Fig. 1 with data available in Table A2. In round 1, the mean HAZ score was the lowest among the older cohort in Ethiopia at −1.56, and the prevalence of stunting was highest in both cohorts in Ethiopia at 41%. Moreover, both India and Ethiopia continued to have higher prevalence of stunting over all survey rounds. However, unlike the other countries, which had only experienced declines in stunting from round 2 to round 3, Ethiopia experienced steady declines in stunting, reaching the same levels as Peru and Vietnam by round 3. This suggested greater catch-up growth in Ethiopia. Demographic characteristics of the sample are presented in Table 2.

**Table 1:** Sample sizes for the paper (% of Young Lives sample)

	Younger cohort	Older cohort
Ethiopia	1,639 (81.99%)	798 (79.80%)
India	1,801 (89.56%)	901 (89.38%)
Peru	1,778 (86.65%)	610 (85.43%)
Vietnam	1,653 (82.65%)	836 (83.60%)
Total	6,871 (85.23%)	3,145 (84.50%)

**Table 2:** Characteristics of the study sample^a^

	Ethiopia	India	Peru	Vietnam
Wealth index	21.14 (3.25)	40.98 (3.24)	44.16 (4.10)	44.49 (3.63)
Sex				
Male	52.11	52.48	50.67	51.27
Female	47.89	47.52	49.33	48.73
Caregiver's education				
None	62.28	62.87	9.12	9.90
Primary	29.53	19.81	44.83	48.87
Secondary or more	8.19	17.32	45.95	41.23
Household size	6.19 (0.12)	5.39 (0.12)	5.55 (0.09)	4.74 (0.09)
Place of residence				
Urban	37.89	25.30	71.22	21.12
Rural	62.11	74.70	28.78	78.88

a All values are means or proportions with standard errors corrected for clustered sampling in parentheses.

**Fig. 2:**
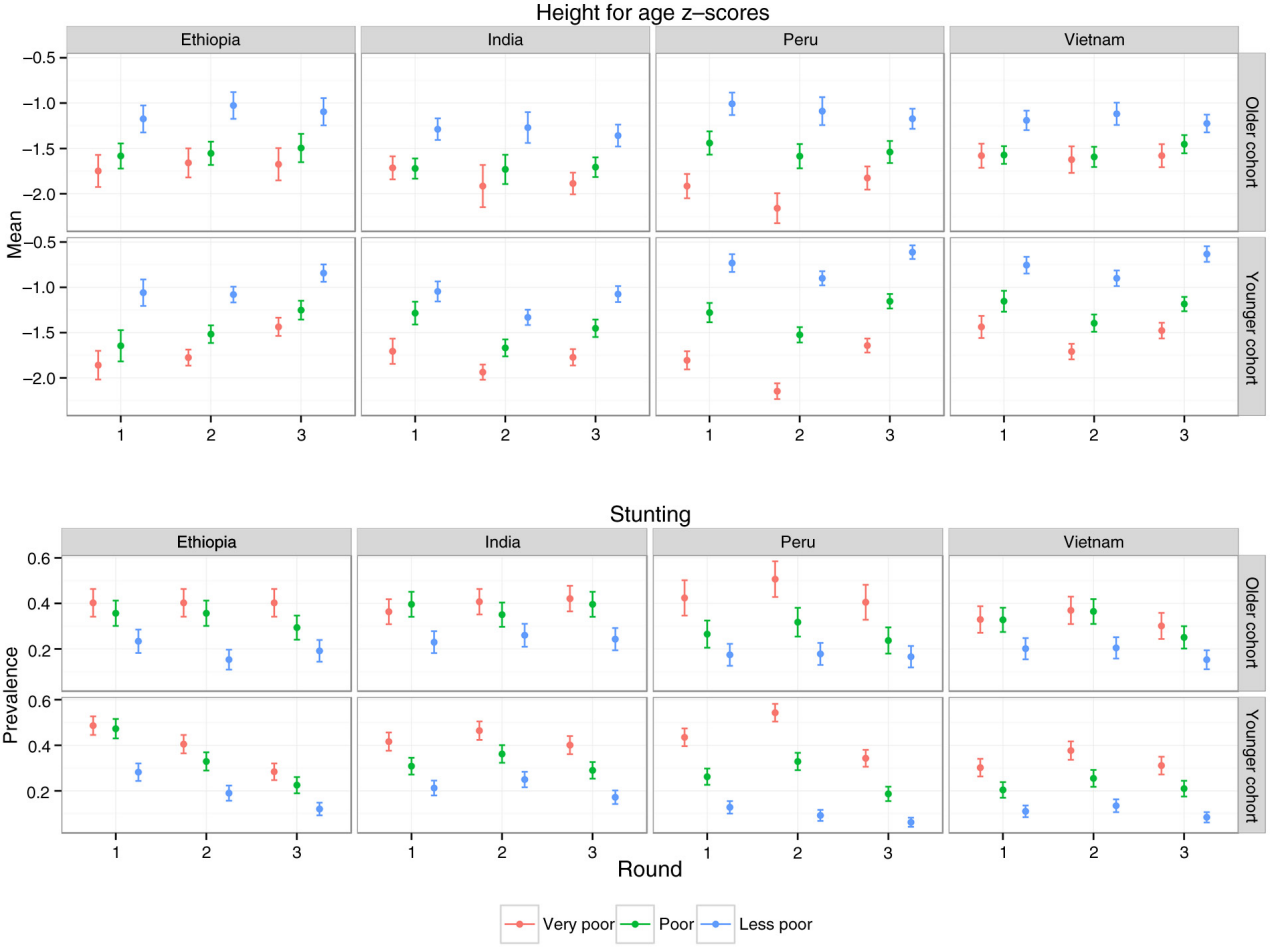
Mean height-for-age z-scores and prevalence of stunting by wealth index category over Young Lives surveys, by country and cohort.

Figure 2 shows that the mean HAZ was higher and the prevalence of stunting was lower in less poor households compared to poor and very poor households. For example, in round 1 in Ethiopia, 28% of children from the less poor group were stunted while 48 and 49% of children for the poor and very poor households were stunted (see Fig. 2). Notably, differences in HAZ and stunting prevalence's between wealth index tertiles persisted over time for both younger and older cohorts.

Table 3 includes results from models examining associations between the baseline wealth index and HAZ. (Results regarding stunting are in Tables A3 and A4.) In the unadjusted models controlling only for survey round, the wealth index was associated with higher HAZ in all countries. In all countries, after controlling for socio-demographic characteristics, associations between the wealth index and HAZ were slightly weaker relative to the estimates in the baseline models. For example, in the unadjusted models for India, a one-unit increase in the wealth index was associated with a 0.011 SD increase in HAZ (SE: 0.0020). In the adjusted model for comparison, the association between the wealth index and HAZ was 0.010 SD (SE: 0.0020). Results from models that included interactions between the wealth index and survey round are presented in Table 4 with some heterogeneity in the magnitude and significance of estimates. For both cohorts in Peru and Vietnam, there were significant differences in the association between baseline wealth index and HAZ by survey round, with greater influences of baseline wealth index in round 2. For the older cohort in Peru, baseline wealth index had a smaller influence in round 3.

**Table 3:** Associations between wealth index at baseline and height-for-age z-scores^a^

		Younger cohort		Older cohort	
	
		Unadjusted	Adjusted^b^	Unadjusted	Adjusted^b^
Ethiopia	Wealth index	0.020*** (0.0044)	0.019*** (0.0042)	0.010** (0.0044)	0.011** (0.0046)
	Round 2	0.064 (0.15)	2.57*** (0.54)	0.087 (0.077)	0.84 (0.62)
	Round 3	0.35** (0.14)	4.60*** (0.89)	0.081 (0.087)	1.37 (1.06)
	Constant	−1.86*** (0.19)	−1.69*** (0.40)	−1.49*** (0.19)	−0.76 (1.37)
	Obs.	1,639	1,639	798	798
India	Wealth index	0.011*** (0.0020)	0.010*** (0.0020)	0.0096*** (0.0022)	0.0071** (0.0030)
	Round 2	−0.30*** (0.11)	0.74** (0.36)	−0.064 (0.062)	0.79 (0.59)
	Round 3	−0.088 (0.11)	1.57*** (0.59)	−0.075* (0.040)	1.29 (1.01)
	Constant	−1.76*** (0.16)	−1.62*** (0.24)	−1.66*** (0.14)	0.37 (1.33)
	Obs.	1,801	1,801	901	901
Peru	Wealth index	0.013*** (0.0014)	0.0098*** (0.0013)	0.010*** (0.0021)	0.0093*** (0.0023)
	Round 2	−0.25*** (0.052)	−0.16 (0.35)	−0.15*** (0.039)	−1.07*** (0.32)
	Round 3	0.14*** (0.048)	0.29 (0.54)	−0.076** (0.034)	−1.56*** (0.49)
	Constant	−1.69*** (0.089)	−1.91*** (0.18)	−1.49*** (0.11)	−2.80*** (0.58)
	Obs.	1,778	1,778	610	610
Vietnam	Wealth index	0.012*** (0.0026)	0.0088*** (0.0025)	0.0086*** (0.0026)	0.0068*** (0.0026)
	Round 2	−0.22*** (0.044)	1.89*** (0.42)	0.0046 (0.021)	0.73*** (0.28)
	Round 3	0.019 (0.048)	3.50*** (0.71)	0.031 (0.045)	1.24*** (0.47)
	Constant	−1.94*** (0.14)	−0.79** (0.36)	−2.08*** (0.15)	0.29 (0.62)
	Obs.	1,653	1,653	836	836

a Results are from ordinary least squares models. Robust standard errors adjusted for clustered sampling are presented in parentheses.

*** *p*<0.01

** *p*<0.05

* *p*<0.1.

Models include survey and sentinel site fixed effects and random effects for individuals.

b Covariates include child's age (in months), child's sex, caregiver's educational attainment (none, primary, secondary or more), household size, and place of residence (rural/urban).

**Table 4:** Associations between wealth index at baseline and height-for-age z-score, by survey round^a^

		Younger cohort	Older cohort
Ethiopia	Wealth index	0.022*** (0.0074)	0.0092** (0.0040)
	Round 2	0.11 (0.26)	0.034 (0.12)
	Round 3	0.43* (0.23)	0.068 (0.13)
	Wealth index*Round 2	−0.0021 (0.0077)	0.0025 (0.0045)
	Wealth index*Round 3	−0.0038 (0.0073)	0.00058 (0.0059)
	Constant	−1.91*** (0.26)	−1.47*** (0.17)
	Obs.	1,639	798
India	Wealth index	0.011*** (0.0024)	0.0073*** (0.0022)
	Round 2	−0.27 (0.18)	−0.25 (0.17)
	Round 3	−0.17 (0.19)	−0.18* (0.090)
	Wealth index*Round 2	0 (0)	0.0044 (0.0030)
	Wealth index*Round 3	−0.00085 (0.0023)	0.0025 (0.0018)
	Constant	0.0020 (0.0031)	−1.56*** (0.13)
	Obs.	1,801	901
Peru	Wealth index	0.012*** (0.0015)	0.010*** (0.0023)
	Round 2	−0.41*** (0.084)	−0.31*** (0.073)
	Round 3	0.12 (0.100)	0.090 (0.058)
	Wealth index*Round 2	0.0035** (0.0016)	0.0035** (0.0014)
	Wealth index*Round 3	0.00042 (0.0017)	−0.0035*** (0.0010)
	Constant	−1.63*** (0.090)	−1.49*** (0.12)
	Obs.	1,778	610
Vietnam	Wealth index	0.0064** (0.0027)	0.0083*** (0.0027)
	Round 2	1.84*** (0.43)	−0.12*** (0.033)
	Round 3	3.54*** (0.72)	0.11 (0.098)
	Wealth index*Round 2	0.0038** (0.0015)	0.0027*** (0.00073)
	Wealth index*Round 3	0.0034 (0.0022)	−0.0018 (0.0020)
	Constant	−0.66* (0.37)	−2.07*** (0.15)
	Obs.	1,653	836

a Results are from ordinary least squares models. Robust standard errors adjusted for clustered sampling are presented in parentheses.

*** *p*<0.01

** *p*<0.05

* *p*<0.1.

Models include survey and sentinel site fixed effects and random effects for individuals. Covariates include child's age (in months), child's sex, caregiver's educational attainment (none, primary, secondary, or more), household size, and place of residence (rural/urban).

### Sensitivity analyses

Tables A5 and A6 show results from additional analyses. Table A5 provides the percentage of children experiencing any changes in wealth index tertile between rounds. Approximately a quarter to half of children experienced changes in wealth index tertiles between rounds with variation between rounds, cohorts, and countries. Table A6 contains results from the adjusted models examining associations between the baseline wealth index and HAZ for children who experienced either upward or downward changes in wealth index to children who did not experience any changes over time. For both cohorts in India, Peru, and Vietnam, there were significant differences in the associations between wealth index tertile and stunting with larger associations for children who experienced no change in wealth index compared to those who did experience changes in wealth index over time. However, despite differences in associations, baseline wealth index affected both groups with higher HAZ for wealthier individuals.

## Discussion

This study demonstrates that household wealth in early life influences physical growth. For example, higher HAZ and lower odds of stunting in later life were predicted among children who lived in wealthier households early on. We observe these associations in all countries, suggesting that living standards matter in a variety of contexts. In addition, the current analysis extends previous work (36), by assessing the lagged effects of early life experiences on child health measured during late childhood for the younger cohort and the beginning of adolescence for the older cohort. We show that even among children who experienced changes in living standards, the influence of early poverty persisted. Thus, our study finds that early experiences of deprivation have lasting influences through infancy, childhood, and adolescence in both cohorts in all four countries.

Another key finding of the study is that associations between household wealth and HAZ are present for both the younger and older cohorts even though baseline wealth index was measured at different life stages. For the younger cohort, baseline wealth index was measured at age 6–18 months; in comparison, for the older cohort, baseline wealth index was measured when the children were 7–8 years old, representing an exposure much later in life. Despite the difference in the timing of measurement, we find that wealth in early life is significantly associated with stunting in both cohorts. Thus, the influences of early poverty on older children, who are beyond the critical period of 1,000 days [beginning at conception and ending at the second birthday during which most growth faltering occurs (44)], suggest that growth faltering may occur among older children (45) and that household living standards in later childhood, beyond the critical period, affect physical growth. One explanation is that the association between baseline wealth index and stunting for the older cohort is confounded by persistent poverty from birth, suggesting that poor living standards at ages seven and eight do not matter. Even for the younger cohort, there is concern that the influence of baseline wealth index is conflated with prolonged experiences of poverty over their life-course.

Comparisons of children who did and did not experience changes in wealth, however, showed only slightly larger associations between baseline wealth index and physical growth for children who experienced no change in living standards as compared to children who experience upward or downward mobility in wealth. Similar associations between baseline wealth index and physical growth for these two groups suggest that the early experience of deprivation may have an independent influence on growth that is not mediated through later life wealth. For the older cohort, we can use persistence in wealth index over the Young Lives follow-up period as a proxy for continuity in living standards before the study's inception. Similar associations between baseline wealth index and physical growth for the group that did and did not experience change in wealth index further emphasizes the importance of living standards at ages seven and eight, refuting the explanation that the influence of baseline wealth index is entirely attributable to persistent, earlier life poverty for the older cohort.

However, inferring causal relationships between wealth and physical growth is complicated by endogeneity problems; there are undoubtedly many unobserved factors that affect both the wealth index and growth even though we included several covariates in order to reduce confounding. In addition, for the members of the older cohort, aged 7–8 at the first survey, it is possible that the baseline wealth index may be affected by poor physical growth. For example, children who are stunted are much more likely to contract infectious diseases (3) and may need hospitalization which requires selling material possessions, thereby lowering the household's wealth index. Although, it is impossible to entirely rule out reverse causation and confounding to obtain a truly exogenous measure of wealth in early life and estimate causal relationships, we were able to assess the downstream influences of an early life exposure on later life outcomes by using longitudinal data from Young Lives. Moreover, although the Young Lives’ sampling strategy does not render the data nationally representative for all countries (24), the data provide a comprehensive understanding of the long-lasting influences of poverty on children in four low- and middle-income countries. Finally, it should be noted that the wealth index may be a poor measure of income or consumption (38). However, it has been widely used as a measure of living standards in other studies including the Demographic and Health Surveys.

## Conclusion

This study contributes new insights about the salient and persistent associations between wealth in early life and physical growth. In particular, it adds to recent work on social determinants of child undernutrition and growth (46–49) and builds on the work in the *Lancet* 2013 Series on maternal and child undernutrition (3) by showing that living standards early in life may influence physical growth in early infancy, childhood, and adolescence. Even among older children, who experience poverty beyond the 1,000 days window, and children living in households with changes in living standards, early experiences of poverty and deprivation have strong and persistent effects later in life. More work should focus on the explanations for the lasting effects of early adversity, particular for children living in low- and middle-income countries who experience a disproportionate burden of both poverty and low physical growth. Our findings are important for policymakers, suggesting that improvements in living standards, particularly those experienced in the early years, may improve child nutrition and child health, even beyond the critical window of 1,000 days.

## Appendix

**Table A1:** Constructing the wealth index

Categories	Elements	Measurement
Housing quality (4 items)	Number of household members per rooms	Continuous variable
	Wall	Dummy variable equal to one if household has brick or plaster wall
	Roof	Dummy variable equal to one if house has sturdy roof
	Floor	Dummy variable equal to one if house has a floor made of finished materials, e.g., cement, tile
Housing quality=Sum of 4 items/4		
Service quality (4 items)	Drinking water	Dummy variable equal to one if house has piped water
	Electricity	Dummy variable equal to one if there is a source of electricity
	Fuel	Dummy variable equal to one if the household uses either electricity, gas, or kerosene
	Sanitation	Dummy variable equal to one if household has pit latrine or flush toilet
Service quality=Sum of 4 items/4		
Consumer durables (11 items)	1. Radio 2. Refrigerator 3. Television 4. Motorcycle/scooter 5. Bicycle 6. Motor vehicle 7. Mobile phone 8. Landline phone 9. Modern bed 10. Table or chair 11. Sofa	Dummy variable for ownership of each of the 11 items
Consumer durables=Sum of 11 items/11		
Wealth index=[Housing quality+Service quality+Consumer durables]/3		

Source: From Ref. [37].

**Table A2:** Prevalence of stunting in Young Lives, by country and cohort

	Ethiopia		India		Peru		Vietnam	
			
	Younger cohort	Older cohort	Younger cohort	Older cohort	Younger cohort	Older cohort	Younger cohort	Older cohort
Height-for-age z-score								
Round 1	−1.53 (0.05)	−1.50 (0.05)	−1.34 (0.04)	−1.58 (0.04)	−1.29 (0.03)	−1.39 (0.04)	−1.11 (0.03)	−1.44 (0.03)
Round 2	−1.47 (0.03)	−1.41 (0.04)	−1.64 (0.03)	−1.64 (0.06)	−1.55 (0.03)	−1.54 (0.05)	−1.33 (0.03)	−1.44 (0.04)
Round 3	−1.18 (0.03)	−1.42 (0.05)	−1.43 (0.03)	−1.65 (0.04)	−1.15 (0.03)	−1.47 (0.04)	−1.09 (0.03)	−1.41 (0.03)
Stunting								
Round 1	0.41 (0.01)	0.33 (0.02)	0.31 (0.01)	0.33 (0.02)	0.28 (0.01)	0.27 (0.02)	0.20 (0.01)	0.28 (0.02)
Round 2	0.31 (0.01)	0.30 (0.02)	0.36 (0.01)	0.34 (0.02)	0.33 (0.01)	0.31 (0.02)	0.25 (0.01)	0.31 (0.02)
Round 3	0.21 (0.01)	0.29 (0.02)	0.29 (0.01)	0.35 (0.02)	0.20 (0.01)	0.25 (0.02)	0.20 (0.01)	0.23 (0.01)
*n*	1,639	798	1,801	901	1,778	610	1,653	836

Standard errors are in parentheses.

**Table A3:** Associations between wealth index at baseline and stunting^a^

		Younger cohort		Older cohort	
	
		Unadjusted	Adjusted^b^	Unadjusted	Adjusted^b^
Ethiopia	Wealth index	0.95*** (0.0091)	0.96*** (0.0084)	0.97*** (0.0081)	0.97*** (0.0090)
	Round 2	0.46*** (0.093)	0.019*** (0.017)	0.77* (0.11)	0.11* (0.13)
	Round 3	0.19*** (0.047)	0.00093*** (0.0013)	0.70** (0.11)	0.027* (0.052)
	Constant	1.24 (1.21)	1.92 (2.08)	0.11 (0.17)	0.018 (0.052)
	Obs.	1,639	1,639	798	798
India	Wealth index	0.96*** (0.0060)	0.97*** (0.0059)	0.97*** (0.0063)	0.98*** (0.0056)
	Round 2	1.51*** (0.21)	0.24* (0.18)	1.09 (0.070)	0.82 (0.98)
	Round 3	0.79* (0.098)	0.041** (0.054)	1.25*** (0.11)	0.79 (1.53)
	Constant	0.48 (0.69)	0.90 (1.35)	0.25 (0.42)	0.089 (0.29)
	Obs.	1,801	1,801	901	901
Peru	Wealth index	0.96*** (0.0056)	0.97*** (0.0049)	0.97*** (0.0073)	0.98*** (0.0079)
	Round 2	1.64*** (0.21)	1.39 (1.23)	1.61*** (0.22)	11.6* (14.9)
	Round 3	0.41*** (0.065)	0.32 (0.44)	0.80* (0.11)	19.5 (37.8)
	Constant	0.83 (0.88)	1.55 (1.73)	0.083 (0.16)	0.75 (2.21)
	Obs.	1,778	1,778	610	610
Vietnam^c^	Wealth index	0.96*** (0.0094)	0.97*** (0.0090)	0.97*** (0.0082)	
	Round 2	1.90*** (0.24)	0.020*** (0.023)	1.34*** (0.13)	
	Round 3	0.94 (0.14)	0.00049*** (0.00100)	0.53*** (0.066)	
	Constant	0.92 (1.22)	0.0076* (0.019)	1.81 (1.59)	
	Obs.	1,653	1,653	836	

a Results are odds ratios from logit models. Robust standard errors adjusted for clustered sampling are presented in parentheses.

*** *p<*0.01

** *p*<0.05

* *p<*0.1.

Models include survey and sentinel site fixed effects and random effects for individuals.

b Covariates include child's age (in months), child's sex, caregiver's educational attainment (none, primary, secondary, or more), household size, and place of residence (rural/urban).

c Due to the low prevalence of stunting among the older cohort in Vietnam, models were not able to converge.

**Table A4:** Associations between wealth index at baseline and stunting, by survey round^a^

		Younger cohort	Older cohort
Ethiopia	Wealth index	0.96*** (0.011)	0.0092** (0.0040)
	Round 2	0.022*** (0.021)	0.034 (0.12)
	Round 3	0.0010*** (0.0015)	0.068 (0.13)
	Wealth index*Round 2	0.99 (0.011)	0.0025 (0.0045)
	Wealth index*Round 3	0.99 (0.013)	0.00058 (0.0059)
	Constant	1.76 (2.00)	−1.47*** (0.17)
	Obs.	1,639	798
India	Wealth index	0.97*** (0.0048)	0.99** (0.0060)
	Round 2	0.25* (0.19)	0.89 (1.03)
	Round 3	0.054** (0.069)	1.01 (1.87)
	Wealth index*Round 2	1.00 (0.0038)	1.00 (0.0039)
	Wealth index*Round 3	0.99** (0.0040)	0.99* (0.0051)
	Constant	0.75 (1.12)	0.061 (0.20)
	Obs.	1,801	901
Peru	Wealth index	0.98*** (0.0048)	0.98** (0.0079)
	Round 2	0.82 (0.69)	7.11* (8.32)
	Round 3	0.039** (0.059)	3.76 (6.60)
	Wealth index*Round 2	0.97*** (0.0060)	0.99*** (0.0045)
	Wealth index*Round 3	0.99 (0.0085)	1.00 (0.0042)
	Constant	0.65 (0.70)	0.080 (0.21)
	Obs.	1,778	610
Vietnam^b^	Wealth index	0.0064** (0.0027)	
	Round 2	1.84*** (0.43)	
	Round 3	3.54*** (0.72)	
	Wealth index*Round 2	0.0038** (0.0015)	
	Wealth index*Round 3	0.0034 (0.0022)	
	Constant	−0.66* (0.37)	
	Obs.	1,653	

a Results are odd ratios from logit models. Robust standard errors adjusted for clustered sampling are presented in parentheses.

*** *p<*0.01

** *p*<0.05

* *p*<0.1.

Models include survey and sentinel site fixed effects and random effects for individuals. Covariates include child's age (in months), child's sex, caregiver's educational attainment (none, primary, secondary, or more), household size, and place of residence (rural/urban).

b Due to the low prevalence of stunting among the older cohort in Vietnam, models were not able to converge.

**Table A5:** Children who experienced changes in wealth index over survey cycles, by country and cohort

	Ethiopia (%)	India (%)	Peru (%)	Vietnam (%)
Younger cohort				
Round 1 to round 2	38.56	32.70	28.91	35.69
Round 2 to round 3	35.75	33.43	37.68	43.25
Older cohort				
Round 1 to round 2	42.61	33.85	25.90	35.41
Round 2 to round 3	36.59	35.18	40.66	40.91

**Table A6:** Associations between height-for-age z-scores and baseline wealth index, controlling for changes in wealth index over Young Lives surveys^a^

		Younger cohort	Older cohort
Ethiopia	Wealth index	0.021*** (0.0044)	0.013* (0.0066)
	Changes in wealth index	0.16 (0.15)	0.080 (0.22)
	Wealth index*Change in wealth index	−0.0060 (0.0047)	0.0041 (0.0058)
	Round 2	2.58*** (0.54)	0.85 (0.63)
	Round 3	4.61*** (0.89)	1.37 (1.07)
	Constant	−1.75*** (0.40)	−0.93 (1.36)
	Obs.	1,639	798
India	Wealth index	0.013*** (0.0019)	0.0099** (0.0040)
	Changes in wealth index	0.24** (0.097)	0.30** (0.15)
	Wealth index*Change in wealth index	−0.0071*** (0.0021)	−0.0059* (0.0032)
	Round 2	0.72** (0.36)	0.81 (0.59)
	Round 3	1.53*** (0.59)	1.32 (1.02)
	Constant	−1.78*** (0.23)	0.23 (1.34)
	Obs.	1,801	901
Peru	Wealth index	0.013*** (0.0014)	0.012*** (0.0024)
	Changes in wealth index	0.35*** (0.062)	0.38*** (0.14)
	Wealth index*Change in wealth index	−0.0066*** (0.0015)	−0.010*** (0.0031)
	Round 2	−0.15 (0.35)	−1.08*** (0.31)
	Round 3	0.31 (0.54)	−1.57*** (0.48)
	Constant	−2.09*** (0.18)	−2.95*** (0.60)
	Obs.	1,778	610
Vietnam	Wealth index	0.0086*** (0.0030)	0.0092*** (0.0027)
	Changes in wealth index	0.00032 (0.12)	0.29* (0.15)
	Wealth index*Change in wealth index	0.00045 (0.0031)	−0.0051* (0.0031)
	Round 2	1.89*** (0.42)	0.74*** (0.27)
	Round 3	3.50*** (0.71)	1.24*** (0.47)
	Constant	−0.80** (0.37)	0.17 (0.61)
	Obs.	1,653	836

a Results are from ordinary least squares. Robust standard errors adjusted for clustered sampling are presented in parentheses.

*** *p*<0.01

** *p*<0.05

* *p*<0.1.

Models include survey and sentinel site fixed effects and random effects for individuals. Covariates include child's age (in months), child's sex, caregiver's educational attainment (none, primary, secondary, or more), household size, and place of residence (rural/urban).
